# Supplementary material for: MetaRibo-Seq measures translation in microbiomes
Source: Nat Commun. 2020 Jun 29;11:3268. doi: 10.1038/s41467-020-17081-z (PMC7324362; doi:10.1038/s41467-020-17081-z)
Supplement: Supplementary file 10 — Supplementary Data 7 [file 41467_2020_17081_MOESM10_ESM.zip › File2/Confidence_High_Taxonomy/204457_out.krona.html]

Javascript must be enabled to view this page.

members
magnitude
magnitudeUnassigned
count
unassigned
taxon
rank

204457\_out

3

3
superkingdom
2

3
phylum
976

200643
3
class

3
order
171549

family
3
1853231

genus
3
283168

species
3
1897010

SRS016335\_contig\_number\_21825SRS047014\_contig\_number\_20449SRS142890\_contig\_number\_33565
